# Differential Secondary Reconstitution of *In Vivo*-Selected Human SCID-Repopulating Cells in NOD/SCID versus NOD/SCID/*γ* chain^null^ Mice

**DOI:** 10.1155/2011/252953

**Published:** 2010-12-26

**Authors:** Shanbao Cai, Haiyan Wang, Barbara Bailey, Jennifer R. Hartwell, Jayne M. Silver, Beth E. Juliar, Anthony L. Sinn, Arthur R. Baluyut, Karen E. Pollok

**Affiliations:** ^1^Section of Pediatric Hematology/Oncology, Department of Pediatrics, Herman B Wells Center for Pediatric Research, Indiana University Simon Cancer Center, The Riley Hospital for Children, 980 West Walnut Street, R3 516, Indianapolis, IN 46202-5525, USA; ^2^Indiana University Simon Cancer Center (IUSCC), Indiana University School of Medicine (IUSM), Indianapolis, IN 46202, USA; ^3^In Vivo Therapeutics Core, IUSCC, Indianapolis, IN 46202, USA; ^4^Biostatistics and Data Management Core, IUSCC, Indianapolis, IN 46202, USA; ^5^Northside Gastroenterology, St. Vincent Hospital, Indianapolis, IN 46260, USA

## Abstract

Humanized bone-marrow xenograft models that can monitor the long-term impact of gene-therapy strategies will help facilitate evaluation of clinical utility. The ability of the murine bone-marrow microenvironment in NOD/SCID versus NOD/SCID/*γ* chain^null^ mice to support long-term engraftment of MGMT^P140K^-transduced human-hematopoietic cells following alkylator-mediated *in vivo* selection was investigated. Mice were transplanted with MGMT^P140K^-transduced CD34^+^ cells and transduced cells selected *in vivo*. At 4 months after transplantation, levels of human-cell engraftment, and MGMT^P140K^-transduced cells in the bone marrow of NOD/SCID versus NSG mice varied slightly in vehicle- and drug-treated mice. In secondary transplants, although equal numbers of MGMT^P140K^-transduced human cells were transplanted, engraftment was significantly higher in NOD/SCID/*γ* chain^null^ mice compared to NOD/SCID mice at 2 months after transplantation. These data indicate that reconstitution of NOD/SCID/*γ* chain^null^ mice with human-hematopoietic cells represents a more promising model in which to test for genotoxicity and efficacy of strategies that focus on manipulation of long-term repopulating cells of human origin.

## 1. Introduction

Development of humanized hematopoietic xenograft models that can monitor both short- and long-term reconstitution of human hematopoietic stem and progenitor cells (HSCs) will be a necessity as hematopoietic stem cell gene therapy trials continue to move forward and show promise in the clinic [1–7]. Clearly the use of small and large animal models has been an intricate component in paving the way for these successes [8–24]. Adverse events, however, directly related to retroviral-mediated insertional mutagenesis in human trials, have been reported in some patients enrolled in gene-therapy clinical trials for severe combined immunodeficiency (SCID)-X1 [25, 26] and chronic granulomatous disease [7, 27]. In addition, as preclinical studies in nonhuman primates, dogs, and mice have progressed over the past decade, development of leukemias most likely linked or initiated by vector-mediated insertional mutagenesis have been reported [28–33]. Therefore, more investigations are needed to understand retroviral-mediated genome instability in addition to the design of new vector systems to prevent this adverse event in human gene-therapy trials. The evaluation of both short- and long-term reconstituting human HSC will be critical in addressing these issues and is the focus of the current study.

In the past*, in vivo* experiments designed to study the engraftment and multilineage differentiation of human HSC were challenging since residual immune reactivity in the animals could result in loss of transplanted human HSC. Additionally, transplant of human HSC into NOD/SCID mice, for example, was typically a short-term 3-4 month assay. With the introduction of new immunodeficient strains such as the NOD/SCID/*γ* chain^null^ mouse (NSG), long-term analysis of engrafted human hematopoietic cells is now a reality and represents a significant improvement over the commonly used NOD/SCID mouse for studies focused on *in vivo* analysis of human HSC function [34]. The NSG mice are deficient in the Interleukin-2 receptor (IL2R) common *γ* chain which is an essential component of multiple cytokine receptors-IL-2, IL-4, IL-7, IL-9, IL-15, and IL-21; this mutation results in a substantial decrease in overall immune function in this mouse strain. In contrast to the NOD/SCID mouse, this additional defect in NSG mice causes a significant block in natural killer-cell development and makes them less susceptible to lymphoma development which allows for an increased life span (NSG = 16 months versus NOD/SCID = 8–10 months). With the increased life span, these mice should be amendable to long-term followup of human HSC function. 

Studies using small and large animal models continue to investigate the consequences of genotoxic stress on retrovirally transduced HSC. A powerful tool for *in-vivo* selection of HSC, a mutant form of O^6^-methylguanine DNA methyltransferase DNA repair protein (MGMT)-MGMT^P140K^-has shown promise in nonhuman primates, mice, and humanized xenograft models. Overexpression of MGMT^P140K^ in HSC, which is resistant to the MGMT inhibitor, O^6^-benzylguanine (6BG) allows for selection and protection of the MGMT^P140K^-transduced HSC following administration of 6BG in combination with alkylators such as BCNU, CCNU, or temozolomide [35]. If MGMT^P140K^ expression is adequate in the HSC, it should also protect the HSC from high-dose alkylator therapy required in some cancer treatments and thereby prevent therapy-induced myelotoxicity. Generation of HSC that efficiently repair DNA damage due to chemotherapy may protect patients from life-threatening cytopenias commonly observed following dose-intensified therapy. A case in point, in recent phase II clinical trials, patients with nitrosourea-resistant gliomas were simultaneously treated with 6BG to deplete MGMT in the cancer cells, followed by treatment with the DNA-damaging agents, BCNU or temozolomide [36, 37]. Although lack of tumor progression was transiently observed in some patients, effective dose-escalation therapy could not be achieved due to severe hematopoietic toxicity. These studies provide clinical proof that strategies protecting HSC during dose-intensified therapy are indeed clearly needed in relapsed patients requiring high-dose alkylator therapy. In addition, expression of MGMT^P140K^ in HSC can be used as a means to preferentially select and amplify small populations of MGMT^P140K^-transduced cells in the bone marrow. However, long-term impact of this treatment in humanized mouse models which include secondary transplantation has not been investigated. To what extent administration of even low doses of chemotherapeutic drugs such as BCNU or TMZ could have on long-term human hematopoiesis is not well understood and secondary malignancies are always a concern. 

Repetitive low-dose treatment for *in vivo* selection of MGMT^P140K^-transduced cells has been successful in mice and large animal models [8, 16]. Numerous transplant studies have convincingly proven that long-term repopulating murine stem cells could be selected *in vivo* with 6BG/BCNU, 6BG/TMZ, or 6BG/CCNU [9, 11, 14, 17, 18, 20, 21, 38–40]. In regards to modeling of this approach with human HSC, we and others previously demonstrated that MGMT^P140K^-transduced SCID-repopulating cells and their progeny could be selected *in vivo *in NOD/SCID mice [19, 24]. Human HSC derived from umbilical cord blood (UCB) or granulocyte colony-stimulating factor (G-CSF)-mobilized peripheral blood (MPB) that expressed MGMT^P140K^ could be selected *in vivo* by nonmyeloablative doses of 6BG and BCNU. Zielske et al. also reported similar results using MGMT^P140K^-transduced UCB in the NOD/SCID xenograft model [24]. Additionally, our laboratory went on to investigate the extent to which MGMT^P140K^-transduced human SCID-repopulating cells and progeny could be protected *in vivo *by MGMT^P140K^ expression during delivery of high-dose alkylator therapy that kills cancer cells. In this study, we compared the outcome of administering a low-dose 6BG/BCNU regimen versus a high-dose regimen in NOD/SCID mice transplanted with MGMT^P140K^-transduced mobilized peripheral blood CD34^+^ cells. We found that, at least in the NOD/SCID xenograft model, when human MPB were transduced with an oncoretroviral vector that expresses MGMT^P140K^, only low numbers of human MPB cells were protected following delivery of the myeloablative regimen and that these cells were limited to mature lymphoid and myeloid cells [10]. In all these studies, NOD/SCID mice were used and analysis of long-term reconstitution in secondary recipient mice was not determined. 

 The objective of our current study was to determine to what degree long-term human SCID-repopulating cells could be selected *in vivo* by alkylator therapy and to compare the levels of selection in primary and secondary NOD/SCID and NSG mice. Our data demonstrate that human hematopoietic cells of multiple lineages were capable of expressing MGMT^P140K^ for at least 4 months in primary recipients and *in vivo*-selected populations while not as robust as nonselected populations, were able to home and engraft in the bone marrow of secondary recipient NSG months for at least an additional 2 months. In contrast to the NOD/SCID xenograft model, the NSG bone-marrow microenvironment appears to allow for optimal reconstitution and feasibility of long-term followup of human hematopoiesis.

## 2. Materials and Methods

### 2.1. Animals

Breeding colonies of NOD.Cg-*Prkdc^scid^* (NOD/SCID) and NOD.Cg-*Prkdc^scid^ IL2rg^tm1Wjl^*/Sz (NSG) mice [34] was established at the Laboratory Animal Research Center and maintained in the *In Vivo *Therapeutics Core at the Indiana University Simon Cancer Center (IUSCC) (Indianapolis, IN). All protocols and were approved by the Institutional Animal Care and Use Committee at the Indiana University School of Medicine.

### 2.2. Isolation of Umbilical Cord Blood (UCB) CD34^+^ Cells

All protocols were approved by Indiana University School of Medicine's Institutional Review Board (IRB) and St. Vincent Hospital's IRB (Indianapolis, IN). Samples of UCB were collected from normal, full-term infants delivered by cesarean section and the CD34^+^ cells isolated using the CD34 MicroBead kit and VarioMACS Separator (Miltenyi Biotech Inc., Auburn, CA) according to the manufacturer's instructions.

### 2.3. Retrovirus Backbones for Expression of MGMT^P140K^ in CD34^+^ Cells

The oncoretroviral vector, SF1-MGMT^P140K^-IRES-EGFP (SF1-P140K) was utilized to coexpress MGMT^P140K^ and EGFP in human CD34^+^ cells and has been described previously [10]. Retroviral vectors were pseudotyped with the gibbon ape leukemia virus envelope (GALV) using the PG13 packaging cell line (American Type Culture Collection, Manassas, Va.) [41]. Titers were initially determined on human erythroleukemia (HEL) cells by limiting-dilution analysis.

### 2.4. Transduction of UCB CD34^+^ Cells

The transductions were done as previously described by our laboratory [10, 19, 42]. The starting cell number prior to prestimulation and transduction was 4 × 10^5^ per mouse transplanted. Isolated CD34^+^ cells were prestimulated at a cell density of 5 × 10^5^ cells per ml in Ex Vivo-10 serum-free medium-containing 1% human serum albumin. The medium was supplemented with Granulocyte-Colony Stimulating Factor (G-CSF), stem cell factor (SCF), and thrombopoietin (TPO) (PeproTech, Rocky Hill, NJ). Each cytokine was used at 100 ng/ml for prestimulation. Nontissue culture 10-cm plates (Falcon, Franklin Lakes, NJ), were coated with 2 *μ*g/cm^2^ Retronectin—(Takara Shuzo, Otsu, Japan) overnight at 4°C. Cells were plated at a concentration of 2 × 10^5^ cells per cm^2^ for transduction. Cells were infected with a 1 : 1 ratio of retrovirus supernatant : complete media with cytokines for 4 hours on 2 consecutive days, with a change to complete medium-containing cytokines for overnight incubation. After the second round of infection, cells were allowed to remain overnight on Retronectin-coated plates with fresh medium and cytokines and then transplanted. The transduction efficiency was determined by flow cytometry on the day of the transplant. For secondary transplants, bone-marrow cells were resuspended at 1 × 10^6^ per ml in Ex Vivo-10 serum-free medium-containing 1% human serum albumin, 100 ng/ml SCF, and 100 ng/ml IL-6 (PeproTech) for 36–48 hours prior to transplantation. 

### 2.5. Transplantation of NOD/SCID and NSG Mice with Human CD34^+^ Cells

Immunodeficient mice were placed on food pellets containing 0.0625% doxycycline for 5–7 days prior to irradiation. Mice were then conditioned with 300-cGy total-body irradiation using a GammaCell 40 (Nordion International Inc., Ontario Canada) equipped with two opposing Cesium-137 sources. UCB CD34^+^ cells were resuspended in IMDM containing 0.2% endotoxin-free bovine serum albumin and injected into the lateral tail vein of each animal.

### 2.6. Chemotherapy Administration

O^6^-benzylguanine (6BG) (Sigma-Aldrich, St Louis, MO) was dissolved in 40% polyethylene glycol-400 (v/v) and 60% saline (v/v). BCNU (Sigma) was dissolved in 10% ethanol (v/v) and 90% normal saline solution (v/v). BCNU was placed on ice and used immediately after reconstitution. One cycle of treatment consisted of 20 mg/kg 6BG followed by 5 mg/kg BCNU one-hour later. Two cycles of treatment were delivered one week apart.

### 2.7. Analysis of Human Cell Engraftment

Mice were sacrificed at 8 weeks after 6BG/BCNU injection and single-cell suspensions of the bone marrow (BM) prepared as previously described [10, 19, 42]. Human cell engraftment measured by human CD45 staining and the proportion of engraftment in various lineages was determined by immunostaining and flow cytometric analysis. Aliquots of 1-2 × 10^5^ cells/tube were stained with various antibodies for 25 minutes at 4°C in complete medium and washed 1 time in PBS containing 1% FBS. All antibodies were titered and used at saturating concentrations. The lack of crossreactivity of human-specific antibodies with murine cells was confirmed in every experiment by staining BM from a nontransplanted mouse with each antibody combination. Cells were stained with Allophycocyanin (APC)-conjugated anti-human-CD45 (anti-HLe-1; Becton Dickinson Immunocytometry, San Jose, CA) alone or in combination with phycoerthyrin (PE)-conjugated anti-human CD33 (anti-Leu-M9; Becton Dickinson). Identical aliquots were stained with APC-conjugated anti-human CD34 (clone 581; PharMingen, San Diego, CA) in combination with anti-human CD19-PE (PharMingen) or anti-human CD38-PE (anti-Leu-17; Becton Dickinson). The forward and right-angle light scatter parameters were used to set the gates for analysis. In experiments where engraftment of human cells was >5%, ~20,000–40,000 events were collected and analyzed. In experiments in which human engraftment was <5% ~200,000 events were collected and analyzed. All samples were acquired and analyzed on a Becton-Dickinson FACSCalibur using CellQuest software (Becton-Dickinson).

### 2.8. Statistical Analysis

The Mann-Whitney *U*-test was utilized to determine statistical significance. Differences between mouse cohorts were considered significant at *P* < .05 using two-sided tests.

## 3. Results

### 3.1. Comparison of Human Hematopoietic Cell Engraftment in NSG versus NOD/SCID Mice

**Figure 1:**
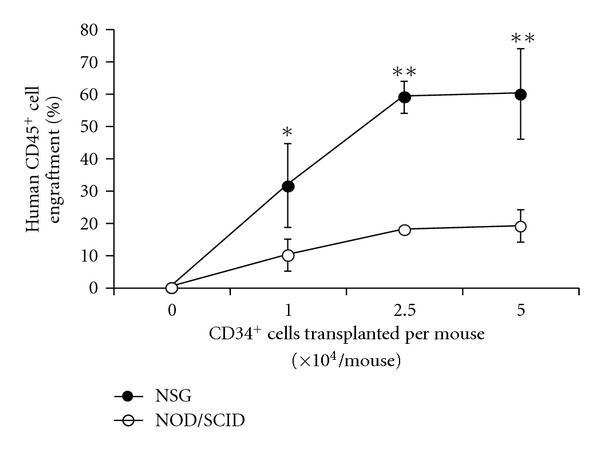
Comparison of human hematopoietic cell engraftment in NSG versus NOD/SCID mice. Nonmanipulated umbilical cord blood CD34^+^ cells (1 × 10^4^–5 × 10^4^ per mouse) were transplanted into sublethally irradiated (300 cGy) NSG or NOD/SCID mice and human cell engraftment in the bone marrow (% human CD45^+^ cells) analyzed 8 weeks later by flow cytometry. Multi-lineage engraftment was similar in both strains (data not shown). *n* = 4 mice per cohort; *NS2 versus NOD/SCID, *P* < .001.

In order to gain a better understanding of the engraftment capabilities of NSG and NOD/SCID mice, we first evaluated the engraftment of nontransduced UCB CD34^+^ cells transplanted into NSG versus NOD/SCID mice. Cohorts of sublethally irradiated NSG and NOD/SCID mice were transplanted with increasing numbers of nonmanipulated, freshly isolated UCB CD34^+^ cells (1.0–5.0 × 10^4^ per mouse). At 2 months after transplantation, the bone marrow was harvested and analyzed for the engraftment of human cells (Figure 1). Human hematopoietic cells engrafted in all NSG and NOD/SCID mice but at higher levels in NSG versus NOD/SCID mice. Engraftment levels in the NSG mice was ~3-fold higher than levels observed in the NOD/SCID mice.

### 3.2. 6BG/BCNU-Mediated In Vivo Selection of MGMT^P140K^-Transduced UCB CD34^+^ Cells in Primary Recipient NOD/SCID and NSG Mice

**Figure 2:**
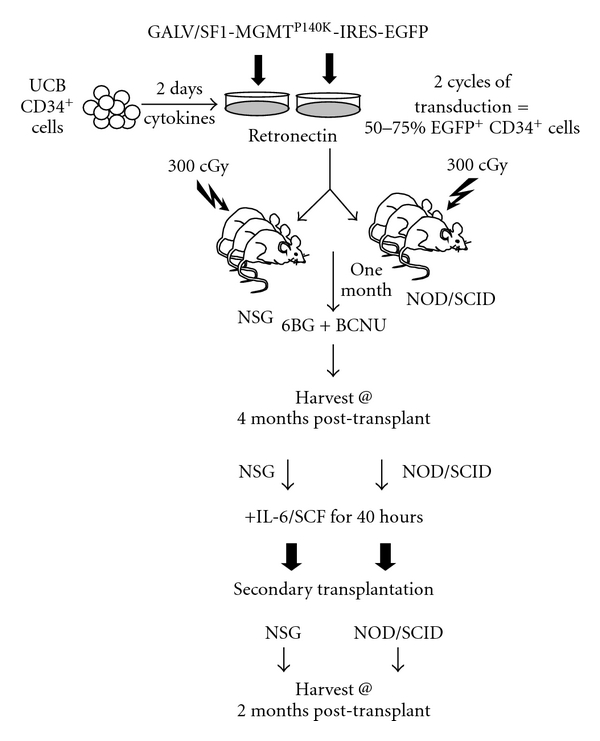
Overview of primary and secondary reconstitution experiments in NSG versus NOD/SCID mice. Human umbilical cord blood CD34^+^ cells were transduced with a GALV-pseudotyped oncoretroviral vector, SF1-MGMT^P140K^-IRES-EGFP (MO1 = 5) and transduced cells transplanted into sublethally irradiated (300 cGy) NSG or NOD/SCID mice. One month after transplantation, cells were selected *in vivo* with 2 cycles of 20 mg/kg 6BG followed one hour later with 5 mg/kg BCNU. At 4 months after transplantation, the bone marrow was harvested and analyzed for the presence of human cells by flow cytometry. Bone marrow cultures were prestimulated in IL-6 and SCF for 48 hours and then equal numbers of human bone-marrow cells were transplanted into secondary recipient NSG and NOD/SCID mice. At 2 months after transplant, the secondary mice were analyzed for the presence of human cells via flow cytometry.

**Figure 3:**
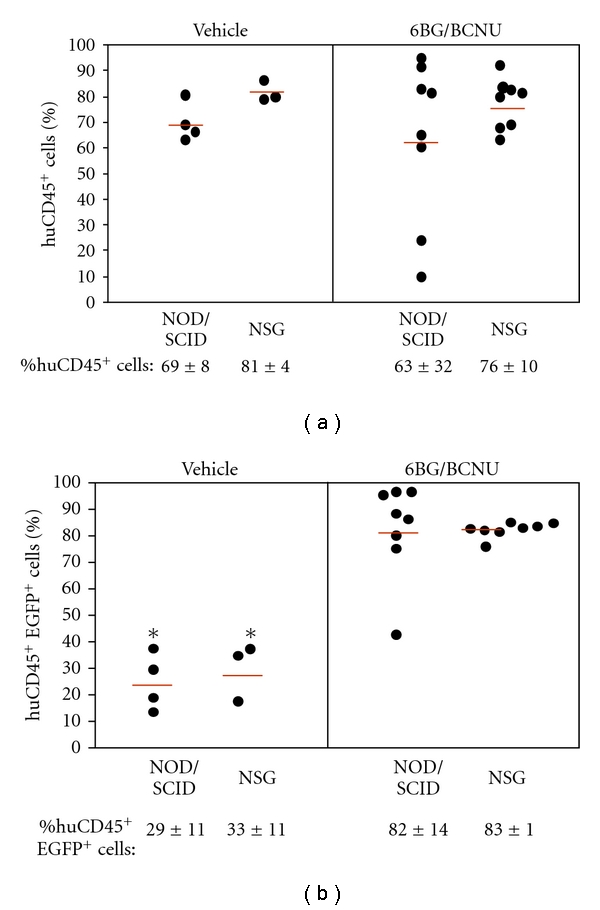
Comparison of *in vivo* selected MGMT^P140K^-transduced human cells in NSG and NOD/SCID mice. The starting cell number was 4 × 10^5^ cells per transplanted mouse. MGMT^P140K^-transduced CD34^+^ cells were transplanted into NS2 and NOD/SCID mice and selected *in vivo* as outlined in Figure 2. The BM was harvested and analyzed for the level of total human cell engraftment (%huCD45^+^ cells) and the level of transduced human cells (%huCD45^+^EGFP^+^) by flow cytometry. The BM cellularity was similar between the NSG and NOD/SCID mice and the differences in total human engraftment in NSG versus NOD/SCID mice were not significant (*P* > .05). The data presented here are from 1 transplant experiment (*n* = 3-4 mice in vehicle-treated cohorts and *n* = 8 mice in 6BG/BCNU-treated cohorts). Similar results were obtained in a second independent transplant experiment.

Human umbilical cord blood CD34^+^ cells were next transduced with a GALV-pseudotyped oncoretroviral vector, SF1-MGMT^P140K^-IRES-EGFP previously shown by our group to express high levels of MGMT^P140K^ in NOD/SCID mice transplanted with MGMT^P140K^-transduced human cells [10]. The transduction efficiency was determined by EGFP expression in bulk CD34^+^ cells and clonogenic cells and was 65% and 60% EGFP^+^, respectively, (data not shown). Equal numbers of transduced cells were transplanted into sublethally irradiated (300 cGy) NSG or NOD/SCID mice (Figure 2). A relatively large number of UCB CD34^+^ cells were transplanted per mouse (4 × 10^5^ total CD34^+^ cells) so sufficient numbers of human cells could be obtained for the secondary transplants following the *in vivo* selection and recovery period. One month after transplantation, mice received 2 cycles administered one week apart consisting of 20 mg/kg 6BG followed one hour later with 5 mg/kg BCNU. At 4 months after transplantation, the bone marrow was harvested and analyzed for the presence of human cells by flow cytometry. Both mouse strains contained high levels of human-cell engraftment in the bone marrow (Figure 3(a)) and *in vivo* selection of transduced cells with 6BG/BCNU was achieved in both mouse strains (Figure 3(b)). However, the overall human engraftment (human CD45^+^ cells) in vehicle-treated NOD/SCID mice was slightly decreased compared to engraftment in vehicle-treated NSG mice (Figure 3(a), vehicle-treated NOD/SCID mice-69% ± 8% versus vehicle-treated NSG mice-81% ± 4). In addition, the variability of human-cell engraftment within the drug-treated NOD/SCID cohort was more pronounced than that seen in the drug-treated NSG cohort (Figure 3(a), drug-treated NOD/SCID mice-63% ± 32% human CD45^+^ cells versus drug-treated NSG mice-76% ± 10% human CD45^+^ cells). In the drug-treated NOD/SCID mice for example, 2/8 exhibited a significant decrease in human chimerism following 6BG/BCNU whereas human-cell engraftment remained more consistent within the NSG drug-treated cohort (Figure 3(a)).

**Figure 4:**
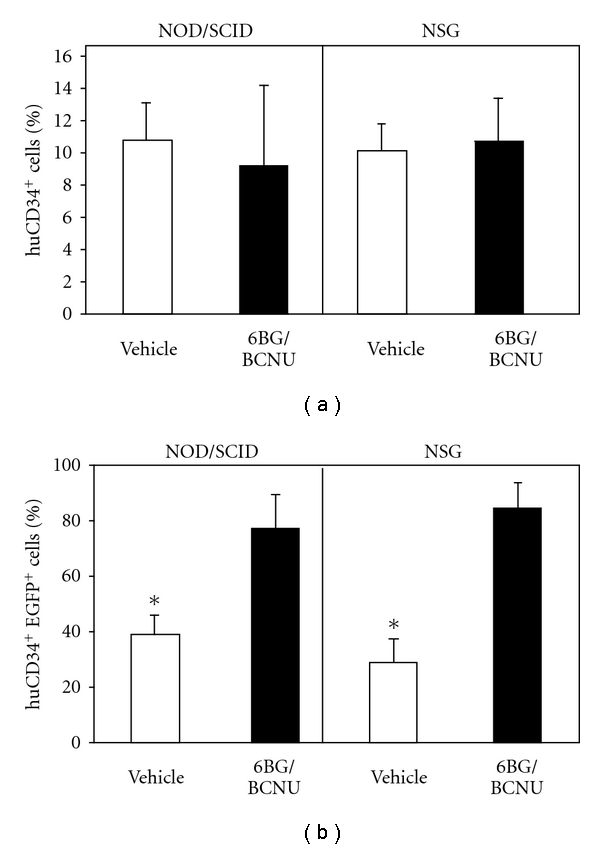
Analysis of human CD34^+^ cells in the bone marrow of primary recipient vehicle- and 6BG/BCNU-treated mice. (a) The percentage of total human CD34^+^ cells and (b) CD34^+^ human cells that are EGFP^+^ (huCD34^+^EGFP^+^) was determined by flow cytometry at 8 weeks after drug treatment. *P < .001, vehicle versus 6BG/BCNU treated.

**Figure 5:**
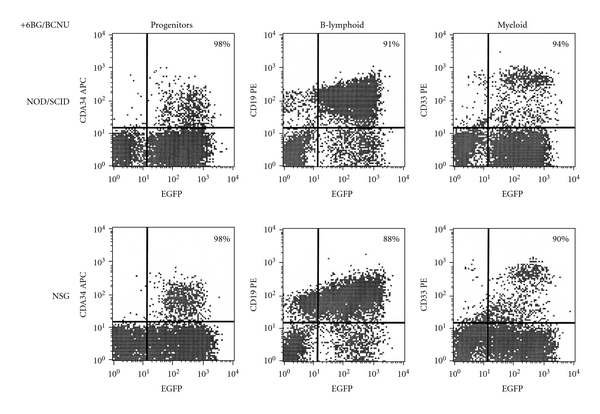
Representative example of human multilineage engraftment in 6BG/BCNU-treated primary recipient mice. The bone marrow was harvested at 8-weeks after drug treatment, and the level of total and transduced human progenitors (CD34^+^), B-lymphoid (CD19^+^), and myeloid (CD33^+^) cells were determined via flow cytometry. The level of transduced cells was similar between NOD/SCID and NSG mice. The percentage in the upper right-hand quadrant represents the percentage of the human progenitor, B-lymphoid, or myeloid cells that are EGFP^+^.

While significant *in vivo* selection of human MGMT^P140K^-transduced cells was clearly evident at 4 months after transplant regardless of the mouse strain, these data indicate that the microenvironment of the NSG mouse which is devoid of murine NK-cell activity may be more amendable for the *in vivo* selection and amplification of transduced human cells. A detailed flow cytometric analysis was also performed to see if any specific cell lineage was preferentially affected by the *in vivo* selection in the mouse strains. While the variability in the percentages of human CD34^+^ cells was larger in the drug-treated NOD/SCID mice than the NSG mice, it did not reach statistical significance (Figure 4(a)). Consistent with the overall *in vivo* selection data presented in Figure 3, there was a significant increase in the *in vivo*-selected CD34^+^ cells in vehicle- versus drug-treated regardless of the mouse strain used indicating a selection and some expansion of the human EGFP^+^, CD34^+^ cells in the bone marrow (Figure 4(b)). The outcome of 6BG/BCNU treatment on multilineage differentiation typically found in the bone marrow of NOD/SCID and NSG mice reconstituted with human cells was next evaluated (Figure 5). In vehicle-treated primary recipient NSG and NOD/SCID mice, similar percentages of total and transduced CD34^+^ progenitor, human B-lymphoid, and myeloid cells were present in the human bone-marrow grafts. Human bone-marrow grafts in vehicle-treated NSG and NOD/SCID mice consisted of 10–15% CD34^+^ progenitor, 65–80% B-lymphoid, and 10–20% myeloid cells, and 20–30% of these lineages expressed EGFP (data not shown). In 6BG/BCNU-treated NSG and NOD/SCID mice, a significant selection of human EGFP^+^ cells was evident in CD34^+^ progenitor, CD19^+^ B-lymphoid and human CD33^+^ myeloid cells with at least 88% of these cell lineages expressing EGFP^+^ at 8 weeks after drug treatment (Figure 5).

### 3.3. Secondary Reconstitution of NOD/SCID and NSG Mice with MGMT^P140K^-Transduced UCB CD34^+^ Cells Derived from NOD/SCID and NSG Mice

**Figure 6:**
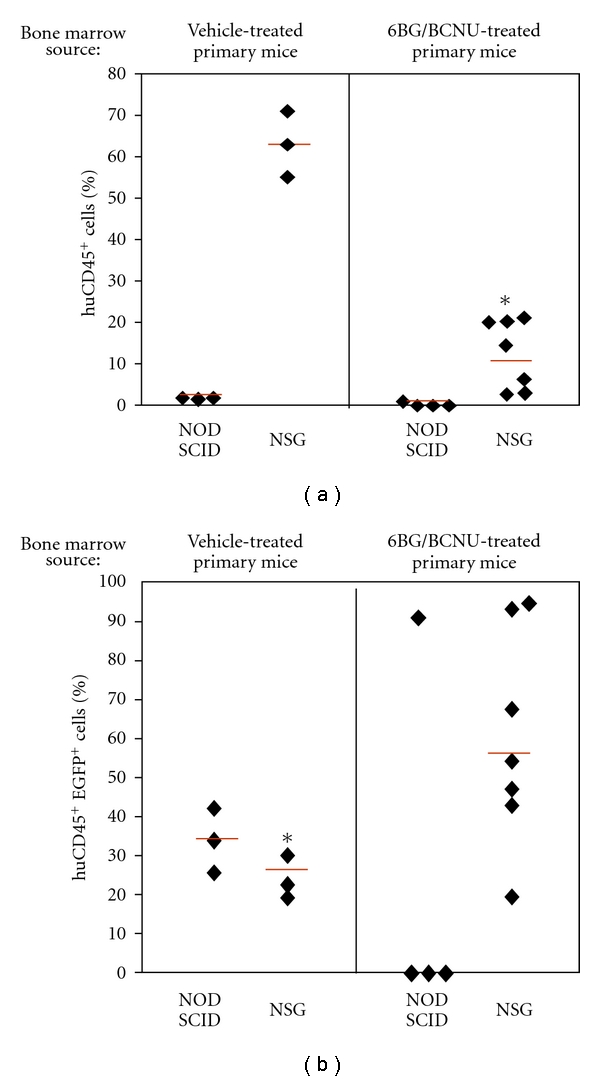
Analysis of human cell engraftment in secondary recipient NOD/SCID and NSG mice. (a) The percentage of total human cells (%huCD45^+^) in the bone marrow and (b) the percentage of the huCD45^+^ cells that are EGFP^+^ (%huCD45^+^EGFP^+^) were analyzed at 8 weeks after transplant into secondary recipient NOD/SCID and NSG mice as outlined in Figure 2. *P < .001, NSG versus NOD/SCID. The date presented are from 1 transplant experiment (*n* = 3 secondary recipient mice transplanted with BM from vehicle-treated cohorts and *n* = 4–8 secondary mice transplanted with BM from 6BG/BCNU-treated cohorts. Similar results were obtained in a second independent transplant experiment.

**Figure 7:**
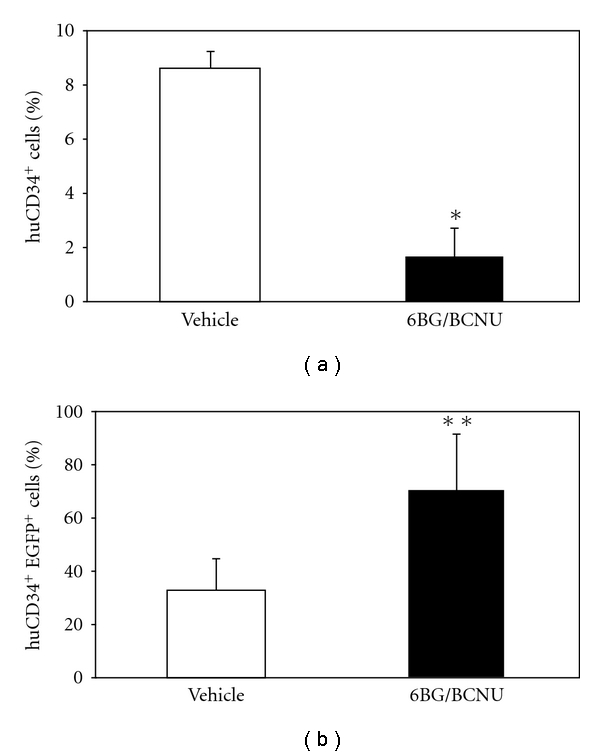
Analysis of human CD34^+^ cells in secondary recipient NSG mice transplanted with BM from vehicle- or 6BG/BCNU-treated mice. (a) The percentage of total human CD34 cells (%huCD34^+^) (b) the percentage of the human CD34^+^ cells that are EGFP^+^ (%huCD34^+^EGFP^+^) in the bone marrow were analyzed at 8 weeks after transplant into secondary recipient NSG mice by flow cytometry. The source of the bone-marrow cells that were transplanted into the secondary recipient mice were derived from vehicle- or 6BG/BCNU-treated mice primary NSG mice. *P < .001, total CD34^+^ cells-vehicle versus 6BG/BCNU treated; *P < .05, transduced CD34^+^ cells-vehicle versus 6BG/BCNU treated.

**Figure 8:**
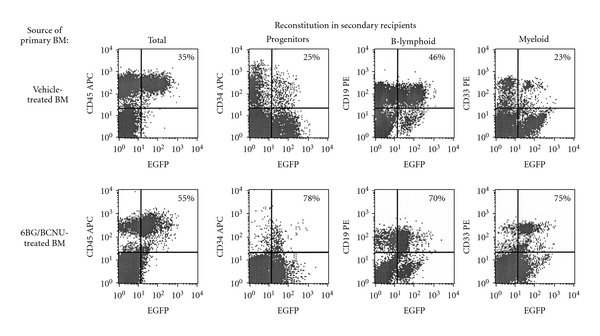
Representative example of multilineage engraftment in secondary recipient NSG mice transplanted with BM from vehicle- or 6BG/BCNU-treated mice. The percentage of total human (CD45^+^) and transduced human progenitors (CD34^+^), B-lymphoid (CD19^+^), and myeloid (CD33^+^) cells in the bone marrow were determined via flow cytometry at 8 weeks after transplant in secondary recipient NSG mice. The percentage in the upper right-hand quadrant represents the percentage of the human cells that are EGFP^+^ in the progenitor, B-lymphoid, or myeloid cell subsets. The source of the bone-marrow cells that were transplanted into the secondary recipient mice were derived from vehicle- or 6BG/BCNU-treated mice primary NSG mice.

Peled et al. originally demonstrated that secondary reconstitution of immunodeficient mice is improved by exposure to IL-6 and SCF prior to retransplantation [43]. The primary mechanism for the enhancement in repopulating ability is increased expression of the CXCR-4 receptor previously shown to play a key role in the homing of transplanted cells. In agreement with these observations, we found that IL-6/SCF prestimulation of human bone-marrow cultures derived from the transplanted NOD/SCID or NSG mice led to an increase or at least maintenance of CXCR-4 expression on the surface of the majority of the human cells (data not shown). Following the 48-hour prestimulation period, 10 × 10^6^ human CD45^+^ cells (vehicle or drug-treated) which contained similar percentages of CD34^+^ cells were transplanted into sublethally irradiated NOD/SCID or NSG mice as outlined in Figure 2. At 2 months after transplantation, the bone marrow of the secondary recipient mice was analyzed for the percentage of total human CD45^+^ cells (Figure 6(a)) and EGFP^+^ human CD45^+^ cells (Figure 6(b)). Even though equivalent numbers of transduced human cells from primary recipient NOD/SCID and NSG mice were transplanted into secondary recipient mice, striking and statistically significant differences in the level of engraftment between the NOD/SCID and the NSG mice was observed (Figure 6(a)). Low levels of engraftment were observed in secondary NOD/SCID mice receiving human cells from vehicle-treated primary NOD/SCID mice and ranged from 2%–4% human CD45^+^ cells with 25–45% of these human hematopoietic cells remaining EGFP^+^. In addition, in NOD/SCID secondary recipient mice transplanted with bone marrow from primary recipient NOD/SCID mice previously drug treated, only 1 of 4 mice showed detectable levels of human cells (1.5% human CD45^+^ cells). In comparison to the NOD/SCID transplants, different levels of human-cell engraftment were found in the secondary transplant experiments using the NSG mice. Human-cell engraftment was detected in all secondary recipient NSG mice (Figure 6), although engraftment levels were different from that previously observed in the primary transplanted NSG mice. Engraftment of NSG mice receiving humanized bone marrow from vehicle-treated mice was 55%–70% human CD45^+^ with 15–30% of the cells transduced. Engraftment of secondary NSG mice receiving humanized bone marrow from drug-treated mice ranged from 4%–18% human CD45^+^ with EGFP^+^ cells ranging from 15%–95%. While a significant increase in the percentage of EGFP^+^CD34^+^ cells was observed in the drug-treated versus the vehicle-treated secondary recipient mice, significant differences were noted in the overall engraftment levels of the CD34^+^ cells in the secondary recipient NSG mice (Figure 7). A significant decrease in the percentage of CD34^+^ cells in the grafts derived from the drug-treated mice compared to grafts derived from vehicle-treated mice was evident and is consistent with decreased long-term SCID-repopulating ability in the secondary recipient NSG mice that received bone marrow from drug-treated primary NSG recipients. The impact of 6BG/BCNU treatment on multilineage differentiation of human cells was next evaluated (Figure 8) and a significant selection of EGFP^+^ human cells was evident in multiple cell lineages in the NSG mice. Increased percentages of EGFP^+^ cells were found in populations of human hematopoietic CD34^+^ progenitor, CD19^+^ B-lymphoid, and CD33^+^ myeloid cells derived from the bone marrow of drug-treated NSG mice indicating that secondary reconstituting SCID-repopulating cells were capable of multilineage differentiation.

## 4. Discussion and Conclusions

Maintenance of genome stability in hematopoietic stem and progenitor cells (HSC) following *ex vivo* manipulation and transplantation will be essential for normal blood-cell development. The objective of this study was to develop a humanized bone-marrow xenograft model to follow long-term reconstitution of genetically modified human HSC. We determined if populations of *in vivo*-selected MGMT^P140K^-transduced human HSC residing in the bone marrow still contained sufficient SCID-repopulating cell activity to reconstitute secondary recipient animals. In this initial study, our focus was to define the basic parameters of the model so that adequate engraftment of HSC in secondary recipients could be achieved. The MGMT^P140K^-transduced human HSC underwent *in vivo* selection in the primary recipient mice one-month after transplant and then the mice were allowed to recover out to 4-months after transplant. We found that while both the NOD/SCID and NSG models adequately supported human HSC reconstitution and *in vivo*-selection in the primary recipient mice, NSG mice were far superior to the NOD/SCID model for secondary reconstitution. It will now be possible to extend the post-transplant time frame, and studies are currently in progress in which genetically modified human HSC are being followed for at least a year after transplantation. 

With basic parameters of the model defined, we are continuing to develop this valuable xenograft model. Studies are now in progress to test lenti- and foamy-viral vector systems to determine if increased numbers of long-term SCID-repopulating cells can be transduced. In addition, the consistency of long-term transgene expression and also analysis of retroviral-insertion sites in primary and secondary transplanted animals in the absence and presence of *in vivo* selection will be evaluated in these long-term studies. 

Since Natural-Killer cell precursor cells require expression of the common *γ* chain protein for development into mature NK cells *in vivo*, lack of this protein will preclude their development. The rejection of human hematopoietic cells by murine NK cells in NOD/SCID mice is most likely the primary reason for decreased engraftment of the human CD34^+^ cells in the NOD/SCID mice but not in the NSG mice. Others have shown previously that the injection of anti-CD122 inhibits NK activity in NOD/SCID mice and improves engraftment in the bone marrow [34, 44–48]. In order to begin to understand the mechanistic differences seen in the engraftment levels of the two mouse strains, we initially looked at the “engraftability” of nonmanipulated CD34^+^ cells in primary recipient NSG versus NOD/SCID mice. Differences in the engraftment of the SCID-repopulating cells in primary NSG versus NOD/SCID recipients mice were apparent when low numbers of human CD34^+^ cells were transplanted. Similar engraftgment results in primary recipient NSG versus NOD/SCID mice have been recently reported by McDermott et al. [44]. It is important to note that our data also demonstrated that when larger numbers of transduced CD34^+^ cells were initially transplanted into primary recipients NOD/SCID or NOD/SCID/*γ* chain^null^ mice, differences in overall human engraftment were evident but were not statistically significant. However, once the chimeric bone-marrow cells were transplanted into secondary recipient animals, engraftment differences in SCID-repopulating activity between NSG and NOD/SCID were statistically significant. 

Bone-marrow cellularity was similar in the NSG and NOD/SCID mouse cohorts indicating that the absolute numbers of EGFP^+^ cells in the bone marrow in the vehicle- and treated-mice in the primary NOD/SCID and NSG mice were also similar. Flow cytometric analyses indicated that the mean fluorescence intensities for EGFP were also similar in the EGFP^+^ populations found in both strains of mice (data not shown). We have previously shown that EGFP expression does correlate with MGMT^P140K^ expression [10]. Therefore, potential differences in transgene expression most likely do not account for the engraftment differences seen between the NOD/SCID and NSG mice.

While the major cellular difference noted in the original paper by Shultz et al. was the lack of murine NK-cell development [34], we cannot rule out that the function of other cell types in the bone-marrow microenvironment could be modulated by lack of the common *γ* chain expression and that this could affect the homing, survival, expansion, and/or differentiation of the human hematopoietic cells. Differential engraftment differences observed in our study are consistent with the hypothesis that the lack of mature murine NK cells in the NSG mice allows for more optimal engraftment, survival, and differentiation of hematopoietic cell subsets since murine NK cells are not present to destroy the human grafts in the NSG mice.

Our data indicate that while we were able to detect human HSC engraftment in secondary NSG mice transplanted with bone marrow from drug-treated primary mice (4%–18% human CD45^+^), it did not approach the levels of reconstitution seen in secondary NSG mice transplanted with bone marrow from vehicle-treated mice (55%–70% human CD45^+^). Furthermore, the percentage of EGFP^+^ cells in secondary NSG mice transplanted with bone marrow from drug-treated primary mice cells varied from 15% to 95%. Transgene expression was more uniform in the drug-treated primary recipient mice (Figure 2(b)). It is not clear at this time whether this decrease in the percent of transduced cells has to do with silencing of the vector and/or decreased SCID-repopulating cell activity in the transduced population. No 6BG/BCNU treatment was administered in the secondary recipient mice and future experiments will also address the outcome of administering *in vivo* selective pressure in the secondary recipient mice. Transplantation of *ex vivo* manipulated HSC into NOD/SCID/*γ* chain^null^ mice represents a feasible model in which to test and validate novel strategies that focus on therapeutic manipulation of long-term repopulating cells from human stem cell sources. Umbilical-cord blood cells were utilized as the transplant source in this study. It will be important to define the long-term SCID-repopulating cell frequency in human bone marrow and G-CSF-mobilized peripheral blood in the NSG mice since these sources will be used in human gene-therapy trials, and typically have lower primary SCID-repopulating cell frequencies than UCB [49], and can be more refractive to transduction than UCB-derived HSC [42]. These data indicate that long-lived SCID-repopulating cells can be transduced with oncoretroviral vectors and selected *in vivo* and offers a promising and unique model in which to assess potential genotoxicity and ultimately optimize *in vivo* selection strategies.

##  Grant Support 

Hope Street Kids Foundation (KEP); the Riley Children's Foundation (SC, HW, and KEP); RO1 CA138798 (KEP).
